# Cognition and renal function: findings from a Brazilian population

**DOI:** 10.1590/2175-8239-JBN-2018-0067

**Published:** 2018-09-13

**Authors:** Viviane Bernardes de Oliveira Chaiben, Thabata Baechtold da Silveira, Murilo Henrique Guedes, João Pedro de Almeida Fernandes, João Henrique Fregadolli Ferreira, Julianna Beltrão, Giovanna Foltran Leal, Lucas Henrique Olandoski Erbano, Natasha Ludmila Bosch, Roberto Pecoits, Thyago Proença de Moraes, Cristina Pellegrino Baena

**Affiliations:** 1 Pontifícia Universidade Católica do Paraná Departamento de Medicina CuritibaPR Brasil Pontifícia Universidade Católica do Paraná, Departamento de Medicina, Curitiba, PR, Brasil.; 2 Pontifícia Universidade Católica do Paraná Escola de Saúde e Biociências PR Brasil Pontifícia Universidade Católica do Paraná, Escola de Saúde e Biociências, PR, Brasil.

**Keywords:** Renal Insufficiency, Cognitive Dysfunction, Dementia, Neuropsychological Tests

## Abstract

**Introduction:**

The prevalence of chronic kidney disease (CKD) is increasing, with a potential impact in the risk of acceleration of dementia. The potential association between glomerular filtration rate (eGFR) and cognitive performance was scarcely studied. The aim of this study was to evaluate cognitive performance levels across different degrees of kidney function.

**Methods:**

We analyzed 240 outpatients in a nephrology service, classified according to eGFR: Advanced (≤ 30ml/min/1.73m^2^), Moderate (30,1ml/min/1.73m^2^ to ≤ 60ml/min/1.73m^2^), and Mild CKD (> 60ml/min/1.73m^2^). Word list memory, Semantic fluency, Mental State Mini Exam and Trail Making Test (TMT) were applied to evaluate cognitive performance. In the TMT, lower scores are associated with better cognition. In linear regression, cognitive function was considered as dependent variables while groups based on eGFR were considered explanatory variables. The group with eGFR > 60ml/min was the reference and models were adjusted for confounding factors.

**Results:**

In our population (n = 240) 64 patients (26.7%) were classified as having advanced, 98(40,8%) moderate, and 78(32,5%) mild. There was no statistical difference among them in MMSE or in the verbal fluency test. However, comparing to mild, patients with advanced CKD presented significantly worse cognitive performance measured by TMTA [50,8s ± 31.1s versus 66,6s ± 35,7s (*p* = 0.016)] and TMTB [92,7s ± 46,2s versus 162,4s ± 35,7s (*p* < 0.001)]. Significantly lower TMTB scores (CI95%) 33,0s (4,5-61,6s) were observed in patients with mild compared to advanced CKD in the multivariate analysis adjusting for age, education, sex, diabetes, and alcohol use.

**Conclusion:**

Advanced CKD is independently associated with poorer cognitive performance measured by an executive performance test compared to mild CKD.

## INTRODUCTION

Chronic kidney disease (CKD) is a public health priority around the world. Its prevalence is approximately 11% in developed countries, and in countries with the worst socioeconomic quartile, people have a 60% higher risk of disease progression.^1^ According to the Global Burden of Disease Study, CKD ranks 24th among the most prevalent diseases in the world, and has increased by 23.8% in the last decade. In Brazil, CKD is estimated to cause 4500-6000 deaths/year. The number of patients in renal replacement therapy between 2000 and 2012 has increased by 3.6% per year. CKD has high morbidity, is associated to mortality, and has elevated social and individual costs. A patient with CKD requires an increased effort to manage the self-care, such as dependency on medical equipment, complex drug prescription, and diet plans; it can also result in a significant psychosocial stress.^2^^-^4

There are several clinical conditions that can lead to increased susceptibility to kidney disease, with arterial hypertension and type 2 diabetes being the main ones. Other risk factors for progression of renal disease are dyslipidemia and smoking.^1^ Cardiovascular risk factors may contribute directly or indirectly to this progression.^5^

Socioeconomic characteristics, such as educational level, may also influence the risk for CKD. According to de BRAZPD Study, a national cohort of peritoneal dialysis in Brazilian patients enrolled from December 2004 to February 2007, there was a large proportion of illiterate patients.^6^ As health literacy is related to educational level, the years of study of those patients may affect negatively the progression of or complications related to CKD.^7^ Several other published studies do not report educational level or illiteracy in their populations.^8^

Another disease with a major health impact is dementia, a clinical syndrome caused by neurodegeneration and characterized by a progressive deterioration in cognitive ability and of an independent life.^9^ Among the different types of dementia, Alzheimer's disease, vascular dementia, Lewy bodies, and frontotemporal dementia are the most common underlying pathologies.^9^ Dementia has a preclinical phase, with evidence of neuropathological lesions beginning 20 years before the appearance of clinically relevant symptoms.^10^ In addition, modifiable risk factors, such as education, physical activity, diabetes, hypertension, obesity, depression, and smoking have already been associated with dementia.^11^

The aging of the population projects an epidemic of dementia cases. According to World Health Organization data, it is estimated that by 2050 the number of people 80 years old or older will quadruple, reaching about 395 million. Moreover, the number of Alzheimer's dementia cases will triple in the same period.^12^ Data from the Global Burden of Disease Study, from 2016, shows that during the last decade, Alzheimer's disease cases increased by 37.7%.^2^

In this sense, common vascular risk factors such as hypertension, diabetes *mellitus*, smoking, dyslipidemia, and cardiovascular disease can affect multiple aspects of cognition.^13^^-^16 The mechanism of brain injury is still unclear, but it is known that a low glomerular filtration rate (e-GFR) can lead to an imbalance in the metabolism of calcium, phosphate, parathyroid hormone, and others that contributes to the acceleration of vascular calcification. Anemia, which can compromise the oxygen supply to the brain, and oxidative stress may contribute to cognitive dysfunction.^4^^,^^17^

The mechanism of injury to nervous tissue may be related to both vascular damage and physiological changes characteristic of the disease as a result of uremia and depression, or even to treatment side effects.^4^ Uremia induces changes in the vascular subendothelium and endothelium, which predisposes patients to accelerated atherosclerosis.^18^

Thus, the aim of this study was to evaluate cognitive function levels across different degrees of kidney function in patients followed in an academic nephrology outpatient clinic.

## METHODS

This is a cross-sectional observational study^19^ carried out at the nephrology outpatient clinic of the Hospital Nossa Senhora da Luz, Curitiba - Brazil, from April to September 2016. The study was approved by the local Ethics Committee.

Patients included in the study attended the nephrology clinic, referred by primary care units or already under follow-up for different clinical conditions, from Curitiba and other regions of the state of Paraná. Patients were recruited consecutively by two nurses who did not know the hypothesis of the study. Exclusion criteria were illiteracy, visual deficit, hearing impairment, use of medications that affect cognition or treatment for active psychiatric illness, such as neuroleptics, antiparkinson, and anticonvulsants.

All those evaluated received orientations about the study and signed the informed consent form prior to inclusion in the study. Next, patients' medical records were analyzed for information about comorbidities, medications of continuous use, blood pressure, recent laboratory tests results, alcohol use, physical activity, and smoking.

Renal function was estimated through serum creatinine and adjusted for age and gender, by the CDK-EPI formula: women = 144 × (Scr/ 0.7)^-0.329^ × 0.993^age^ and men = 144 × (Scr/ 0.7)^-0.411^ × 0.993^age^.^5^ The calculation was performed without considering race as a variable, due to the characteristics of the Brazilian black population that differs from the American, which was used as basis for the definition of the CKD EPI calculation.^3^

### COGNITIVE TESTS

Patients were submitted to cognitive tests, which assessed executive cognitive function. Namely, verbal fluency (number of animals cited by the participant in 1 minute), immediate memory (number of words mentioned and remembered immediately by the participant), and guidance, memory, attention, and understanding through the Mini Mental State Examination (MMSE),^20^ Trail Making Test part A and B (TMT A and B) (Appendix 1). In the TMT part A, the circles are numbered from 1 to 25 and the patient must draw lines to connect the numbers in ascending order. In Part B, the circles include numbers from 1 to 13 and letters from A to L; as in Part A, the patient draws lines to connect the circles in an ascending pattern, but with the added task of alternating between the numbers and letters. Scores represent the time required to complete the test, such that lower scores imply shorter time.^20^ Since depression is the disease that most generates diagnostic confusion with cognitive deficit,^9^ it was evaluated through the Geriatric Depression Scale.^21^ The tests were applied in a silent office, by a team previously trained by a psychologist and supervised by the investigator, and lasted about 30 minutes.

### STATISTICAL ANALYSIS

Considering the effect of glomerular function on cognition reported in other studies (R^2^ = 0.10),^22^ with a power of 80%, and an alpha level of 5%, the size of the sample was estimated to be 232 patients.

Continuous variables are presented as means and standard deviations, or as medians or interquartile ranges, as indicated. Categorical variables are presented as proportions. The comparisons were done by analysis of variance (ANOVA) and the proportions were compared by the Chi-square test. For variables without a normal distribution, the Kruskal Wallis test was performed. The glomerular filtration rate variable was categorized in 3 categories: category 1, e-GFR less or equal to 30 mL/min/1.73m^2^; category 2, e-GFR between 30.1 and 60 mL/min/1.73m^2^; and category 3, e-GFR above 60.1 mL/min/1.73m^2^.

In multilinear regression models, continuous variables of cognitive function were considered dependent variables and the renal function category was considered the explanatory variable, with the e-GFR above 60.1 mL/min/1.73m^2^ as the reference.

Variables that had significance in the univariate analysis were used as co-variables, in addition to gender, age, education, diabetes, smoking, and alcohol. The level of significance was determined at 5% and the analyses were performed with the IBM SPSS Statistics 20 statistical package.

## RESULTS

**Figure 1:**
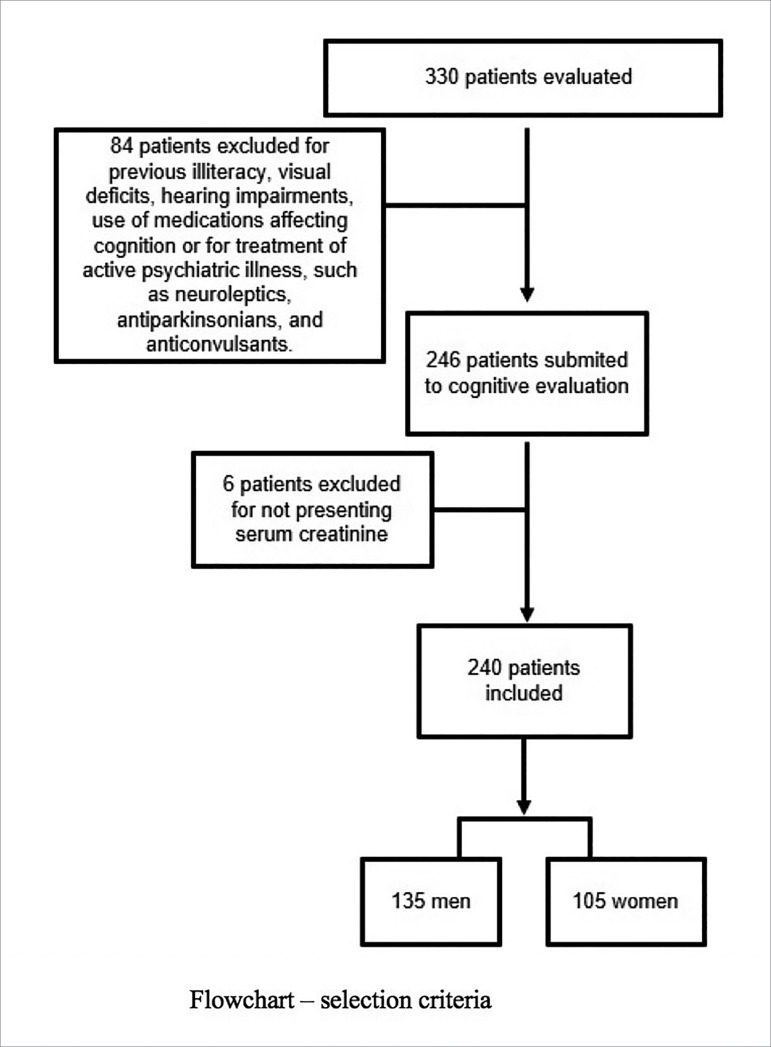
Flow Chart of the participants.

Out of the 330 patients evaluated, 84 were excluded based on the exclusion criteria (illiteracy, visual or auditory deficit, use of medications). Of the 246 patients submitted to cognitive evaluation, 6 were excluded for not presenting serum creatinine, which made it impossible to evaluate renal function (Figure 1).

A total of 240 patients were included in the study and subsequently divided according to e-GFR. The characteristics of the sample according to glomerular function categories are presented in Table 1. Sex, age, hypertension, alcohol use, dyslipidemia, and diabetes differed significantly among renal function categories.

**Table 1:** Clinical characteristics of patients according to the eGFR (n = 240; men = 135; women = 105)

		Variable		
	> 60 (n = 78; 32.5%)	30.1 - 60 (n = 98; 40.8%)	≤ 30 (n = 64; 26.7%)	*p*-value*
Male	32 (41.0)	66 (67.3)	37 (57.8)	0.002
Age (years)	48.9 ± 14.0	63.1 ± 15.1	63.4 ± 12.6	< 0.001
Years of study				0.007
Up to 4	20 (25.6)	45 (45.9)	34(53.1)	
5 a 8	26 (33.3)	18 (18.4)	13(20.3)	
9 or more	32 (41.0)	35 (35.7)	17(26.6)	
Married	49 (62.8)	63 (64.3)	33 (51,6)	0.234
Smoking	35 (44.9)	47 (48.0)	40 (62.5)	0.086
Alcohol use	24 (30.8)	16 (16.3)	10 (15.6)	0.031
Sleep disturbance	56 (71.8)	67 (68.4)	51 (79.7)	0.284
Practices physical activity	36 (46.2)	42 (42.9)	24 (37.5)	0.581
Systolic Blood Pressure	134.8 ± 24.3	136.1 ± 22.0	145.5 ± 28.2	0.035
Cognitive deficit (MMSE)	34 (44.2)	44 (44.9)	26 (40.6)	0.858
Diabetes	12 (15.4)	28 (28.6)	31 (48.4)	< 0.001
Hypertension	56 (87.5)	81 (90.0)	59 (93.6)	0.498
Dyslipidemia	29 (37.2)	58 (59.2)	35 (54.7)	0.011
Use of anticonvulsants	2 (2.6)	1 (1.0)	1 (1.6)	-

Results reported as mean ± standard deviation or as frequency (percentage)

* one-way ANOVA or Chi-square test; p < 0.05

Information on cognition is presented in Table 2. Patients in the worst renal function category presented the worst cognitive results in the executive function tests TMT A (*p* = 0.016) and TMT B (*p* < 0.001) and in the anterograde memory evaluation (*p* = 0.049). There was no statistical difference in depression among the 3 categories in MMSE or in the verbal fluency test.

**Table 2:** Results of the cognitive tests among patients in different stages according to GFR

Variable		*p*-value*		
	> 60	30.1 - 60	≤ 30	
Mini Mental State Exam (MMSE) (n = 238)				0.301
N	78	97	64	
Median	26.5	25	26	
IR	5	5	6	
Geriatric Depression Scale - 15 (n = 236)				0.753
N	77	96	63	
Median	4	3	3	
IR	3	5	3	
Anterograde Memory Test (n = 237)				0.049
N	77	97	63	
Median	6	6	6	
IR	1	2	2	
Verbal Fluency Test (n = 237)				0.120
N	77	97	63	
Median	16	15	15	
IR	8	8	5	
Time of TMT A (n = 227) (in seconds)				0.016
N	77	91	59	
Mean	50.8	60.9	66.6	
SD	31.1	31.6	35.7	
Time of TMT B (n = 146) (in seconds)				< 0.001
N	57	55	34	
Mean	92.7	128.6	162.4	
SD	46.2	66.5	97.0	

IR: interquartile range; SD: standard deviation

* One-way ANOVA or non-parametric Kruskal-Wallis test; *p* < 0.05.

TMT A: *Trail Making* test part A TMT B: *Trail Making* test part B

The results of the multiple linear regression analysis of the cognitive function tests are shown in Table 3. Results of the executive function tests (TMT A and B), in addition to models in which the values were adjusted for age, years of study, gender, diabetes, cognitive deficit, and alcohol use are presented. Independent of age, years of study, gender, diabetes, education and alcohol use, those in the worst glomerular filtration rate category presented poorer performance on TMT B test results. In comparison to category 1, category 3 takes 69.7 (40, 99) seconds longer (*p* value < 0.001) to execute the test. In category 2 the difference is 35.8 (10.2, 61.4) seconds (*p* value = 0.006) compared to category 1.

**Table 3:** Linear coefficients for Trail Making Test part A and part B (in seconds), considering the eGFR categories

Test*	Category	Crude model		Model 1		Model 2	
		β (CI95%)	*p*-value	β (CI95%)	*p*-value	β (CI95%)	*p*-value
TMT A	> 60 (ref)						
	30.1 - 60	10.1 (0.20 - 20.1)	0.046	-0.1 (-9.8 - 9.6)	0.991	0.1 (-9.1 - 9.2)	0.986
	≤ 30	15.9 (4,8 - 26.9)	0.005	4.4 (-6.1 - 14.9)	0.405	3.2 (-7.0 - 13.3)	0.540
TMT B	> 60 (ref)						
	30.1 - 60	35.8 (10.3 - 61.4)	0.006	20.6 (-5.4 - 46.5)	0.119	6.3 (-19.1 - 31.7)	0.624
	≤ 30	69.7 (40.4 - 99.0)	< 0.001	48.6 (19.8 - 77.4)	0.001	33.0 (4.5 - 61.6)	0.024

B (95%CI): estimated coefficient and confidence interval of 95%

* Time to complete the test in seconds

TMT A: *Trail Making* test part A TMT B: *Trail Making* test part B Crude model 1: adjusted for age, years of study, and gender Model 2 - TMT A: adjusted for age, years of study, gender, diabetes, and cognitive deficit Model 2 - TMT B: adjusted for age, years of study, gender, diabetes, cognitive deficit and alcohol use

## DISCUSSION

Even though both CKD and dementia share common risk factors, the independent impact of CKD severity on dementia has not been investigated in Brazilian patients. The main finding of this study was that advanced CKD is independently associated with poorer cognitive performance measured by an executive performance test.

Additionally, our findings show that, among patients with renal dysfunction, the prevalence of cognitive dysfunction increases linearly as e-GFR declines, and this association is independent of age, years of study, gender, diabetes, and use of alcohol. The other cognitive function tests, anterograde memory and verbal fluency test, did not present differences in the studied sample.

Several recent studies have explored the risk of cognitive impairment in patients with renal dysfunction. Tamura *et al*., in the CRIC study, evaluated 3591 patients through the MMSE and demonstrated that an e-GFR lower than 30 mL/min/1,73m^2^ is associated with a 47% increase in cognitive decline, regardless of disease and vascular risk factors. However, the association is not significant after adjusting for hemoglobin levels, demonstrating the influence of anemia in this dysfunction.^14^ These same authors in another study aimed at evaluating the association between cognitive decline and CKD progression, prospectively analyzed 3883 patients with a baseline cognitive impairment assessed by the MMSE for a period of 6.1 years. They reported no association between the variables analyzed.^23^ These findings are in accordance with ours' lack of association when using MMSE to evaluate cognition.

Lee *et al*. studied an elderly Japanese population of 4686 regarding the relationship between cognitive decline and CKD in patients without dementia. Using the functional evaluation tool of the National Center for Geriatrics and Gerontology, they also demonstrated that lower eGFRs are associated with worse cognitive function, both in the attention and processing speed domains.^24^

Through the application of different tests, and especially due to the sensitivity of the TMT B, in which the visual search, scanning, processing speed, mental flexibility, and executive functions are evaluated, it is possible to detect premature cognitive function loss.^4^^,^^25^ Selinger *et al*. demonstrated that not only do patients with renal dysfunction have a worse performance in verbal analysis and/ or visual memory tests, but also have a faster decline of these functions with age.^26^

It is known that cognitive function declines with age, but the rate of this decline varies greatly among individuals.^27^ Among patients with renal dysfunction, studies have reported a lower average in cognitive performance when compared to the population with preserved renal function.

Contrarily to our findings, Helmer *et al*. in 2011 did not find an association between glomerular filtration rate and cognitive decline or dementia, but demonstrated that cognitive function is worse in more severe degrees of renal dysfunction.^28^

The association between cognitive decline, especially of the executive function, and renal dysfunction could be explained by the endothelial dysfunction that occurs in the early stages of kidney disease, in patients with cardiovascular risk factors, and in those with cognitive impairment. However, studies show that after adjusting for such factors, the association between the two remains significant,^29^ pointing to an independent effect.

The pathophysiology of cognitive decline in patients with renal dysfunction is unclear. However, one can hypothesize that characteristics of the affected cognitive domains resemble vascular dementia, since patients present attention and executive function deficit, with difficulties in motor performance and information processing.^30^

Neurological disorders may be associated with impaired renal function, such as oxidative stress, anemia, presence of metabolic toxins, or to vascular risk factors such as hypertension, diabetes mellitus, smoking, dyslipidemia, and cardiovascular disease. The vascular pathology seems to be related to the involvement of small vessels, leading to endothelial dysfunction accompanied by inflammation. Ikram *et al*. in 2008 demonstrated that altered renal function is associated with cerebral small vessel vascular disease markers, demonstrated through magnetic resonance imaging of patients, and that this occurs independently of cardiovascular risk factors.^13^^-^16^,^^28^^,^^31^

Our results are in line with these previous ones, since our analyzes were controlled for cardiovascular risk factors, such as age, gender, and alcohol use, which were not controlled for by most previous studies. Additionally, our results extend the current evidence to a population in a developing country, where dementia is projected to reach alarming levels in the coming decades.^10^

Among limitations is the study design, which limited the inference of temporality between cognitive function and renal function. Another limitation was not determining hemoglobin levels, and consequently anemia, to demonstrate the relationship of cognition with this parameter.^14^ It can also be considered that patients attending health services are not representative of the general population. However, the results found here have clinical implications and might support funding for dementia preventive measures and for improvement of treatment strategies for our population under treatment for kidney disease.

These results highlight the need to screen for cognitive decline in individuals with CKD in order to detect possible difficulties in treatment and to ensure that patients and their family caregivers can understand and, therefore, guarantee treatment adherence. A prospective analysis of the association between cognitive decline and decreased glomerular filtration rate may elucidate the impact on clinical outcomes in this population.

In conclusion, our study shows that advanced CKD is independently associated with poorer cognitive performance, especially in executive function, regardless of age, gender, years of study, and alcohol use in a population attended at a reference nephrology outpatient clinic.

## SUPPLEMENTARY MATERIAL

The following online material is available for this article:

Appendix 1
