# Supplementary material for: Cognition and renal function: findings from a Brazilian population
Source: J Bras Nefrol. 2018 Sep 13;41(2):200–7. doi: 10.1590/2175-8239-JBN-2018-0067 (PMC6699443; doi:10.1590/2175-8239-JBN-2018-0067)

Supplementary Material to "Cognition and renal function: findings from a Brazilian population"

Appendix 1

Verbal Fluency Test

Verbal fluency: Animals

- Tell the patient: "I'm going to set the clock for 1 minute, and I want you to tell me the names of all the animals you can remember at that time, which is the name of any animal or animal."
- Make sure the person understands the instruction. If necessary, exemplify: "If I were asking for fruit names, you would speak apple, orange, etc, understood?"
- If you have understood the instructions, say "it may begin." Examine times 1 minute and record patient responses at that time. If there are different names for the same animal according to sex (bull, cow, etc.), consider the two answers.

The final score corresponds to the total number of animals remembered in 1 minute

Immediate Memory Test

Memory - anterograde memory				
Say, "I'm going to give you a name and an address and I'd like you to repeat after me, we'll do it three times, so you'll have the chance to learn them, I'll ask you later."Punctuate only the third attempt				Score (0-7)
Renato Moreira Bela<br>Vista Street 73<br>Santarém<br>Pará	1st attempt<br>……… …………… …….<br>………… ……. ………<br>………<br>………	2nd attempt<br>……… …………… …….<br>………… …… ………<br>………<br>………	3rd attempt<br>……… ……………<br>……. ……… ………<br>…………<br>…..……	

Mental State Mini Exam

Name:____
Schooling: _________________________ marital status:____________________________
Appraiser:____
Date: ____/_____/______

Guidance (10 points)
What is the approximate time? .....……...( )
What day of the week is it? ......……....... ....( )
What is the date today? .....………........... ....( )
What is the month? .... ……..( )
What is the year? .....……........... ....( )
Where are we now? .....……........... ....( )
What is this place? .....……........... ....( )
In what district are we or what is the address here? ....( )
In which town are we? .....……..... ....( )
In which state are we? .....… ....( )

Registration (3 points) .....……........... ....( )
Repeat the following words: CAR, VASE, BRICK

Attention and calculation (5 points) ....( )
Subtract: 100-7 = 93-7 = 86-7 = 79-7 = 72-7 = 65

Remote memory (3 points) .... ………..( )
Can you remember the 3 words you have just said?

Language
Naming 2 objects (2 points).... ....( )
Watch and pen REPEAT (1 point) " neither here nor there nor there" …..( )
Stage command (3 points) "Take this piece of paper with your
right hand, fold it in half, and put it on the floor” …..( )
Writing a complete sentence (1 point) …..( )
Write a sentence that makes sense Reading and obey (1 point) ………...( )
Close your eyes Copy the diagram (1 point) …..( )
Copy two pentagons with an intersection …..( )
SCORE (__/30)

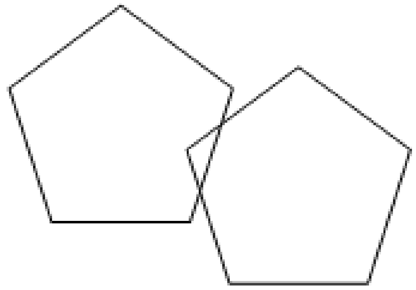

Trail Making Test Part A

Trail Making Test Part A – *SAMPLE*

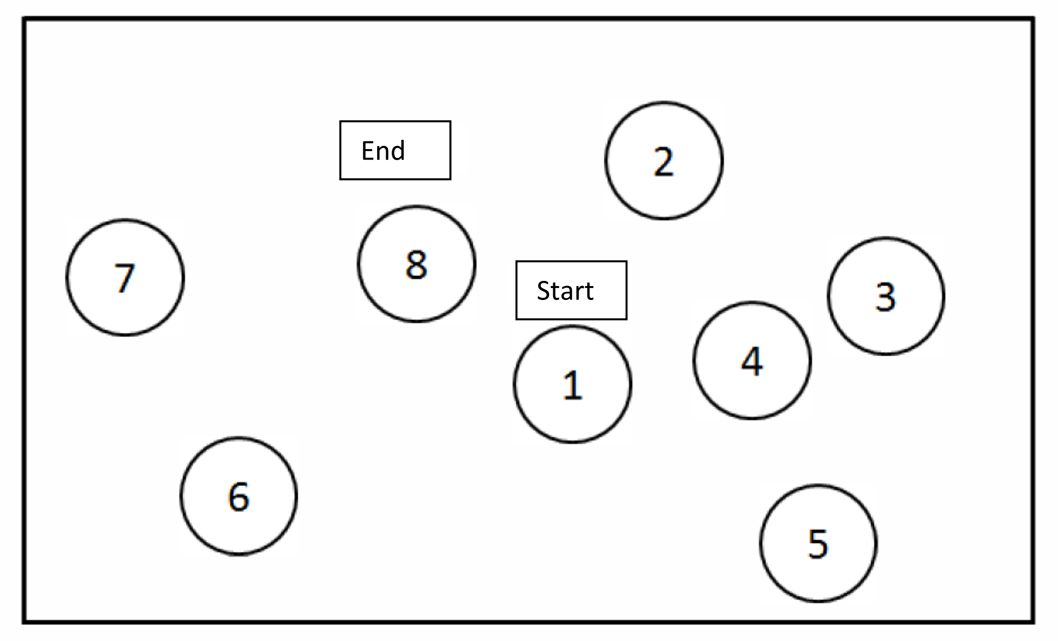

Trail Making Test Part A

Name:____ Date:____________

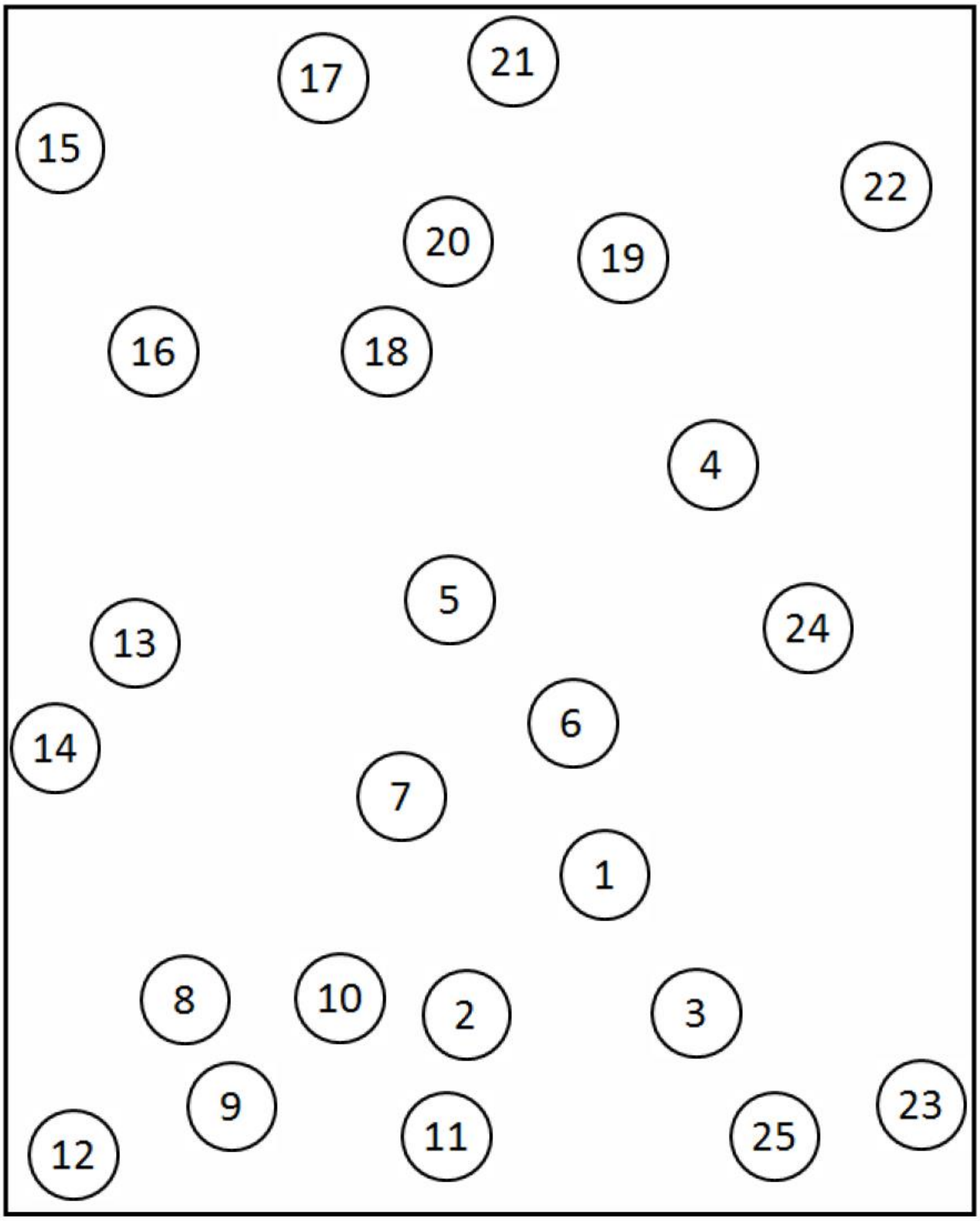

Trail Making Test Part B

Trail Making Test Part B – *SAMPLE*

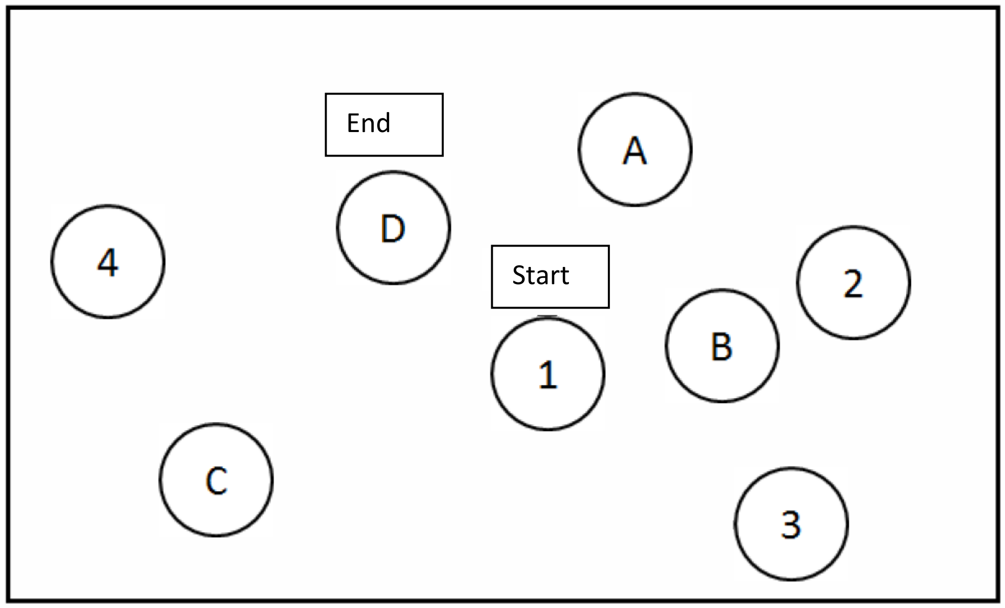

Trail Making Test Part B

Name:____ Date:____________

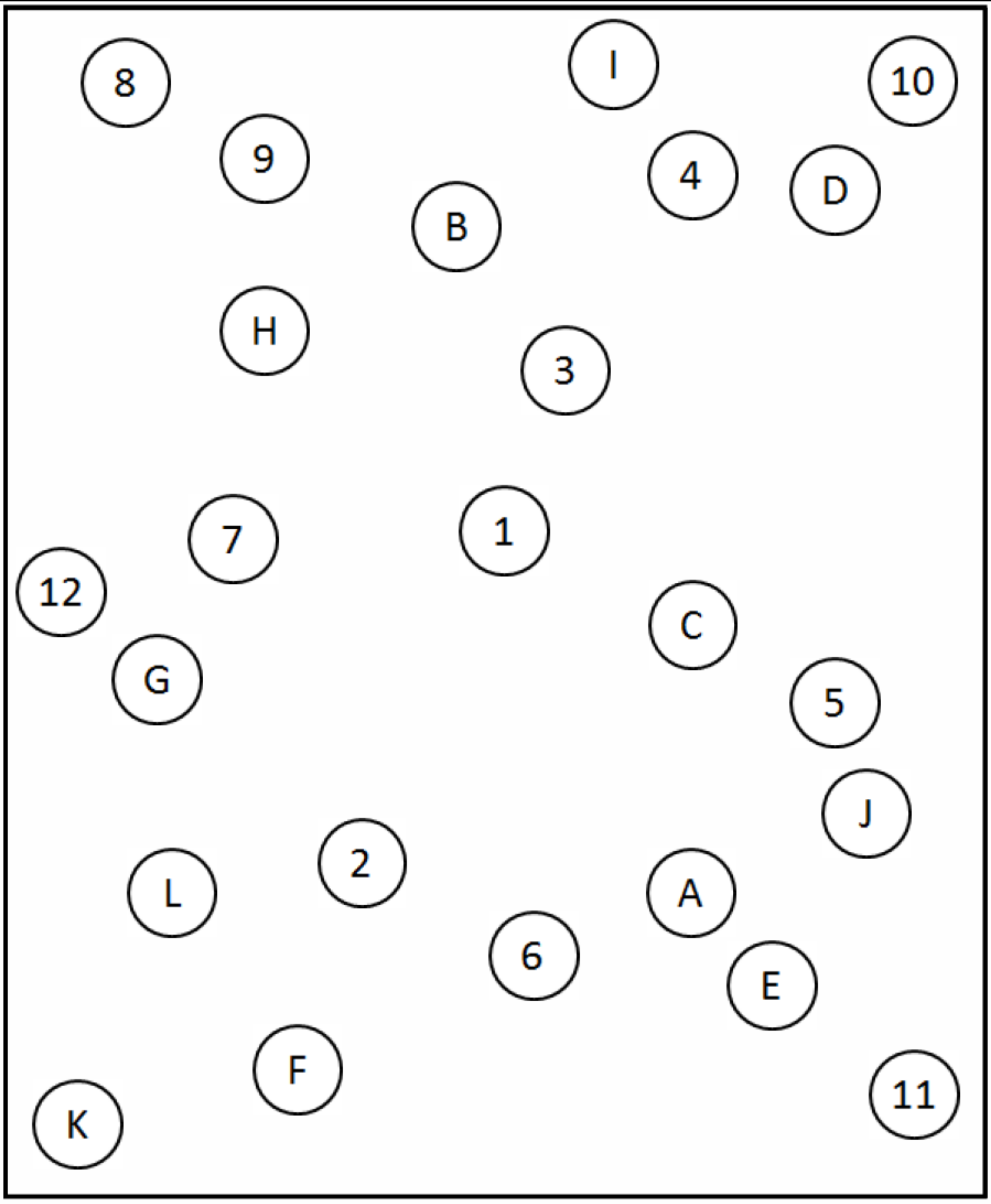

Supplement: Appendix 1 [file 2175-8239-jbn-2018-0067-suppl01.pdf]
